# Impact of femoral mediolateral diameter on femoral component selection during total knee arthroplasty and its effect on postoperative knee function and pain: a retrospective cohort study

**DOI:** 10.3389/fsurg.2026.1885142

**Published:** 2026-07-30

**Authors:** Xingyu Liu, Xiaojin Bai, Peiwen Zhao, Qiang Zheng, Bingyan Xiang

**Affiliations:** Department of Joint Surgery, The First People’s Hospital of Zunyi (The Third Affiliated Hospital of Zunyi Medical University), Zunyi City, Guizhou, China

**Keywords:** Asian population, aspect ratio, femoral component, mediolateral diameter, osteoarthritis, total knee arthroplasty

## Abstract

**Objective:**

To investigate the influence of different intraoperative reference dimensions on femoral component selection during total knee arthroplasty (TKA) and to compare the postoperative knee function, pain, and radiographic outcomes between two sizing strategies when the femoral mediolateral (ML) and anteroposterior (AP) diameters are discrepant.

**Methods:**

This retrospective cohort study included 86 patients (86 knees) with primary knee osteoarthritis who underwent TKA. Patients were allocated to two groups based on the intraoperative femoral sizing reference: Group A (*n* = 52), in which the component was selected strictly according to the femoral AP diameter; and Group B (*n* = 34), in which, on the basis of AP evaluation, a component one to two sizes larger was chosen to improve mediolateral coverage when the ML/AP ratio exceeded 1.13. Operative time, intraoperative and postoperative blood loss, Hospital for Special Surgery (HSS) knee score, Visual Analogue Scale (VAS) for pain, and knee range of motion (ROM) were recorded preoperatively and at 1 week, 3 months, and 1 year postoperatively.

**Results:**

Both groups achieved significant improvement in HSS score, VAS score, and knee ROM at each follow-up time point compared with preoperative values (all *P* < 0.001). Group B showed a smaller knee ROM (96.74° ± 4.19° vs. 100.83° ± 5.50°, *P* < 0.001) and higher VAS score (3.35 ± 0.60 vs. 2.90 ± 0.66, *P* = 0.002) at 1 week, and similar trends persisted at 3 months. Operative time, intraoperative blood loss, and postoperative drainage were comparable between groups (all *P* > 0.05). Radiographic shadowing of the uncovered distal femoral condyle region was significantly more frequent in Group A than in Group B (65.38% vs. 20.59%, *χ*^2^ = 16.54, *P* < 0.001). No deep infection, aseptic loosening, or periprosthetic fracture occurred in either group during follow-up.

**Conclusion:**

TKA remains an effective treatment for end-stage knee osteoarthritis. In Asian patients with an ML/AP ratio greater than 1.13, choosing a femoral component one to two sizes larger than that predicted by the AP reference appropriately increases distal mediolateral coverage without adversely affecting one-year functional outcomes or anterior knee pain, and significantly reduces radiographic shadowing in the uncovered distal femoral area. This strategy deserves consideration in routine clinical practice.

## 1 Introduction

Knee osteoarthritis (KOA) is one of the most common degenerative joint diseases worldwide and a leading cause of chronic pain and disability in the aging population (1, 2). Global estimates indicate that the prevalence and burden of KOA have risen substantially over the past three decades, driven largely by population aging and the obesity epidemic (1, 3). Total knee arthroplasty (TKA) is widely accepted as the most effective surgical intervention for end-stage KOA when conservative management fails, providing reliable pain relief, improved knee function, and high long-term survivorship (2, 4). Contemporary single-centre cohort data report implant survivorship approaching 94% at 10-year follow-up for well-performed primary TKA with modern posterior-stabilized (PS) designs (4), and patient satisfaction rates of approximately 89%–90% have been reported in large contemporary series (5, 6).

Despite these favourable outcomes, a substantial minority of patients continue to experience residual pain, anterior knee pain, or dissatisfaction after TKA (6, 7). Among the many technical factors that influence postoperative outcomes, femoral component sizing and fit have attracted increasing attention (8–10). Optimal sizing requires balancing three competing goals: adequate bone coverage to transmit load across the prosthesis–bone interface, avoidance of mediolateral (ML) overhang that can irritate soft tissues and cause pain, and preservation of proper flexion–extension gaps and posterior condylar offset (PCO) to maintain kinematics (8, 9, 11). Morphometric studies have consistently shown that off-the-shelf femoral components, the majority of which were originally developed from Caucasian anatomical databases, may provide sub-optimal fit for a significant proportion of patients when used globally (8, 10).

Because total knee arthroplasty technology and prosthetic design were originally developed predominantly in North America and Europe, most commercially available femoral components exhibit a relatively constrained mediolateral-to-anteroposterior aspect ratio, and many implant systems provide only one mediolateral width for each anteroposterior size (8, 12). However, anthropometric studies have shown that distal femoral dimensions and ML/AP ratios vary across populations, and that conventional implant geometries may not adequately reproduce the native anatomy of Asian and other non-Caucasian patients (13–15).

However, morphometric investigations performed in East Asian populations, including Chinese, Korean and Japanese cohorts, have repeatedly demonstrated that distal femoral dimensions differ meaningfully from those of Caucasian populations, with Asian knees generally exhibiting a smaller AP dimension for a given ML width and therefore a higher ML/AP aspect ratio (10, 16, 17). In a CT-based morphometric study of 406 osteoarthritic knees from southwestern China, the resected distal femoral aspect ratio deviated clearly from that offered by six commonly used TKA systems in China, with under-sizing in the ML dimension being the most frequent pattern (10). Similar trends have been reported in other Asian populations, suggesting that a considerable proportion of Asian TKA candidates have an ML/AP ratio greater than 1.13 (16, 17).

The current dominant sizing strategy in mainland China is posterior (AP) referencing, in which the femoral component is chosen to match the AP diameter so as to preserve the flexion gap and avoid anterior femoral over-stuffing (11, 18). While this strategy theoretically minimizes patellofemoral overstuffing and anterior knee pain, it may leave the distal medial and lateral femoral bone surfaces uncovered in patients with a high ML/AP ratio, raising concerns about long-term peri-implant bone remodelling, stress shielding, and periprosthetic fracture risk (8, 9). Conversely, upsizing the component to cover more of the ML dimension risks mediolateral overhang, increased patellofemoral pressure, and anterior knee pain (19, 20). The trade-offs between these two strategies in the Asian population remain incompletely defined, especially regarding the short- and mid-term impact on functional recovery, VAS pain score, ROM, and radiographic bone behaviour in the uncovered region.

Several anthropometric investigations have demonstrated that East Asian knees often have distal femoral dimensions and ML/AP aspect ratios that differ significantly from those of Caucasian populations. For example, Japanese and other East Asian patients were reported to have mean ML/AP aspect ratios of ∼1.25–1.31 on CT measurements, which are generally higher than typical Western values (14), and Indian knees have been shown to exhibit larger ML/AP ratios compared to Hispanic and Caucasian knees (13, 15). These population-specific differences suggest that conventional implant aspect ratios may not optimally fit all patients, especially in Asian cohorts.

The aim of the present retrospective cohort study was therefore to compare the early-to-mid term clinical and radiographic outcomes of two intraoperative femoral sizing strategies—strict AP referencing vs. ML-biased up-sizing—in Chinese patients with knee osteoarthritis undergoing primary TKA with the same PS implant system. We hypothesized that, in patients with an ML/AP ratio greater than 1.13, up-sizing the femoral component by one to two sizes to improve ML coverage would not significantly compromise functional recovery or increase anterior knee pain at the one-year follow-up, while reducing the occurrence of radiographic shadowing in the uncovered distal femoral bone area.

## 2 Materials and methods

### 2.1 Study design and setting

This was a single-centre retrospective cohort study conducted at the Department of Joint Surgery, the First People's Hospital of Zunyi (the Third Affiliated Hospital of Zunyi Medical University). Consecutive patients with primary knee osteoarthritis who underwent unilateral primary TKA between January 2022 and January 2023 were screened. The study protocol was approved by the hospital institutional review board. Written Informed consent was waived because of the retrospective nature of the study, with all data anonymized for privacy protection. The reporting of this study conforms to the STROBE statement for observational research. All surgical procedures were performed by the same senior joint surgery team using a standardized technique. Clinical records, operative notes, perioperative laboratory data, and imaging studies (plain radiographs, full-length standing weight-bearing lower-extremity films, and thin-slice CT with three-dimensional reconstruction) were retrospectively reviewed through the hospital electronic medical record system.

### 2.2 Inclusion and exclusion criteria

**Figure 1:**
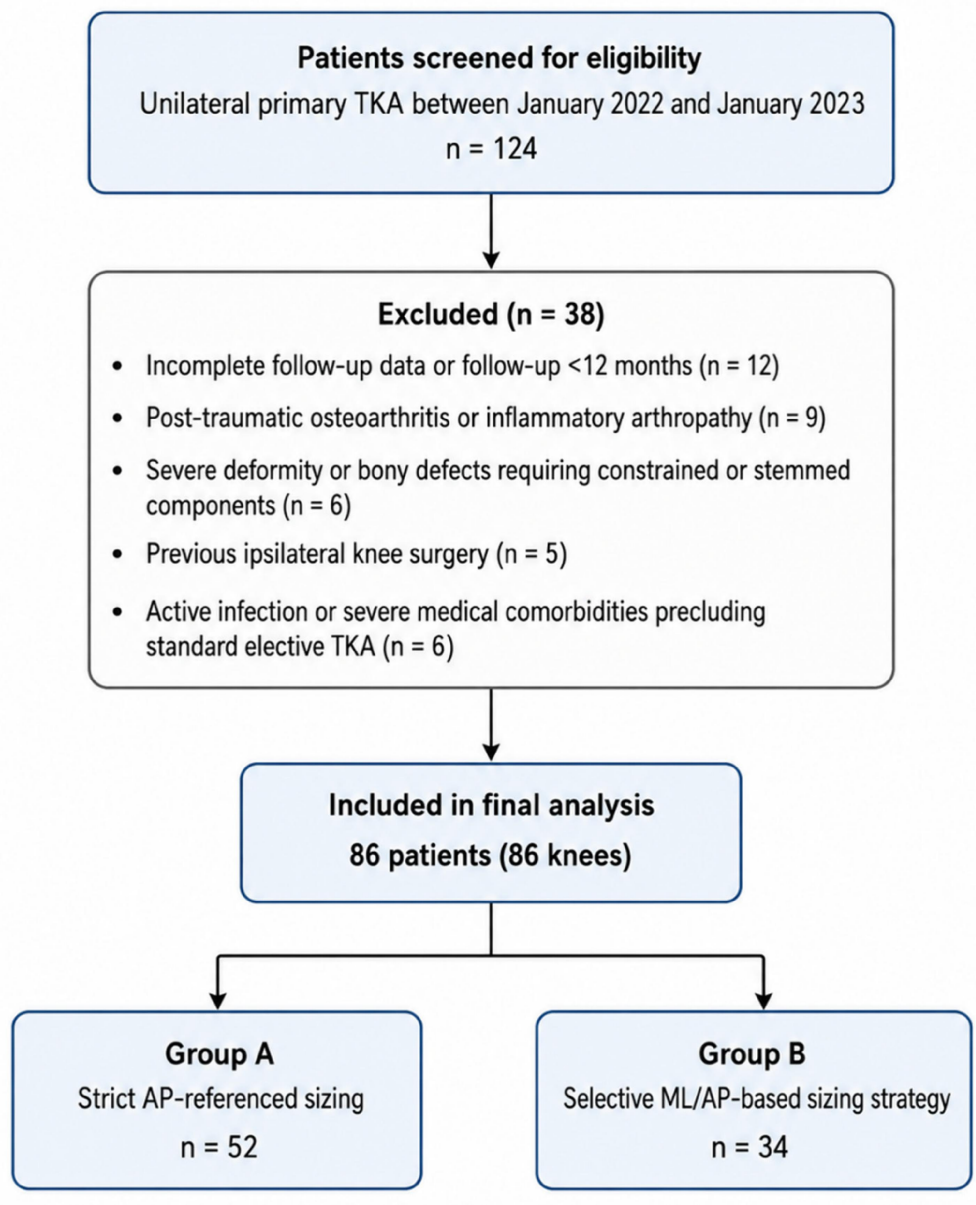
Flowchart of patient selection and grouping. A total of 124 patients were screened for eligibility. After exclusion of 38 patients based on the predefined criteria, 86 patients were included in the final analysis, with 52 assigned to Group A (strict AP-referenced sizing) and 34 assigned to Group B (selective ML/AP-based sizing strategy).

Patients were eligible if they met the American College of Rheumatology diagnostic criteria for knee osteoarthritis and were scheduled for primary TKA at our institution during the study period. Specifically, inclusion criteria were as follows: (1) a clinical and radiographic diagnosis of primary knee osteoarthritis with varus deformity less than 20° on full-length standing radiographs and no significant bony defect of the tibia or femur; (2) intact medial and lateral collateral ligament function so that a constrained liner was not required; and (3) absence of extra-articular deformity of the femur or tibia that would require osteotomy or a stemmed implant. Exclusion criteria were as follows: (1) active or latent infection of the knee joint or elsewhere in the body; (2) a recent episode of severe cardiovascular or cerebrovascular disease, gastrointestinal perforation or haemorrhage, or any condition that contraindicated elective surgery; (3) medical comorbidities that rendered the patient unable to tolerate general anaesthesia or prolonged surgery; (4) post-traumatic osteoarthritis, inflammatory arthropathy, or previous ipsilateral knee arthroplasty; and (5) incomplete follow-up data or follow-up of less than 12 months. A total of 124 consecutive patients (124 knees) who underwent unilateral primary TKA between January 2022 and January 2023 were initially screened. Of these, 38 patients were excluded, including 12 with incomplete follow-up data or follow-up of less than 12 months, 9 with post-traumatic osteoarthritis or inflammatory arthropathy, 6 with severe deformity or bony defects requiring constrained or stemmed components, 5 with previous ipsilateral knee surgery, and 6 with active infection or severe medical comorbidities precluding standard elective TKA. After applying these criteria, 86 patients (86 knees) were included in the final analysis. The patient selection and grouping process is shown in Figure 1.

### 2.3 Preoperative evaluation and imaging

**Figure 2:**
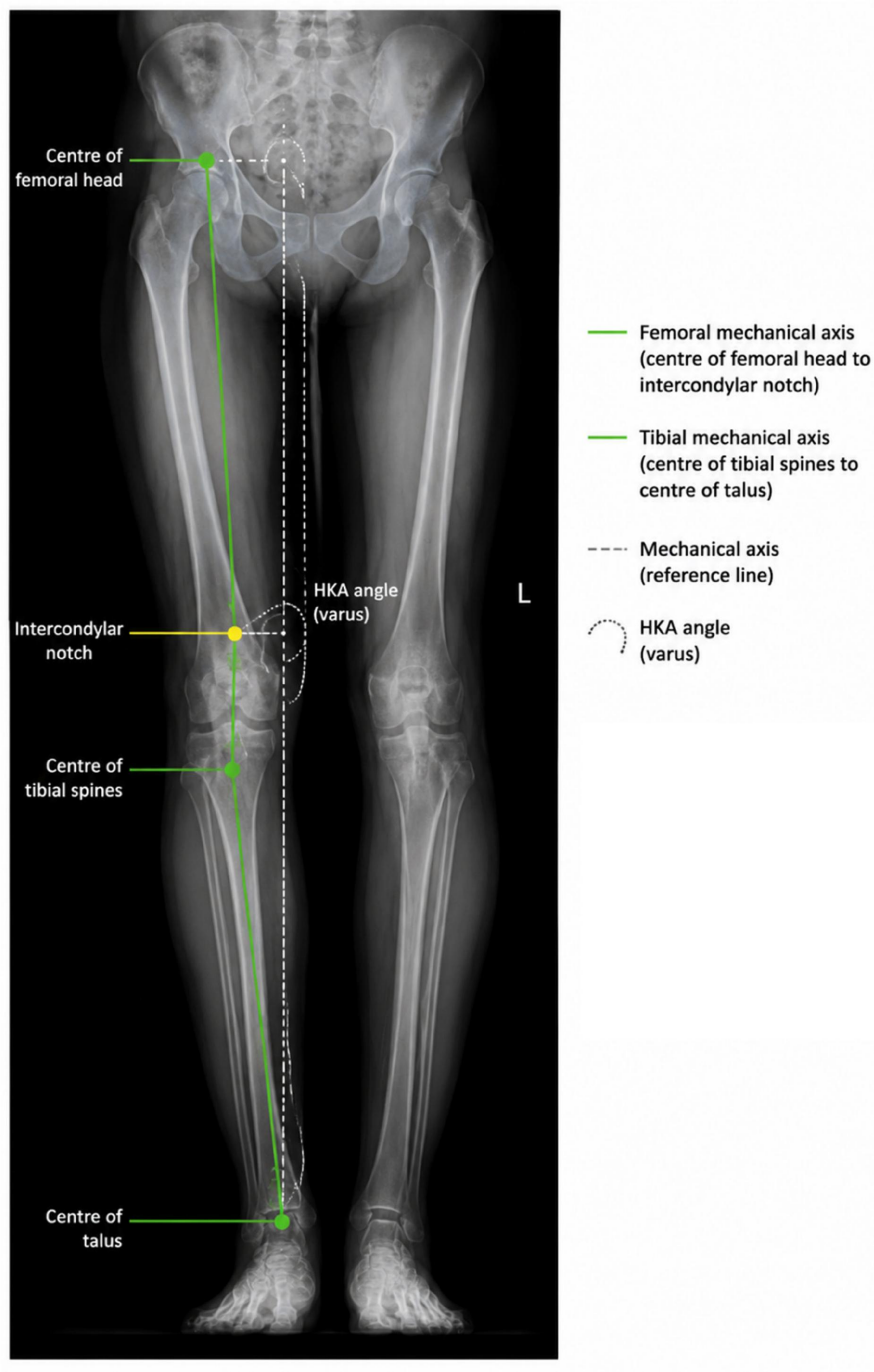
Preoperative full-length weight-bearing anteroposterior radiograph of the lower extremities used to measure the mechanical axis, hip–knee–ankle (HKA) angle, and preoperative varus deformity. The femoral mechanical axis was defined by the line from the centre of the femoral head to the intercondylar notch, and the tibial mechanical axis by the line from the centre of the tibial spines to the centre of the talus.

**Figure 3:**
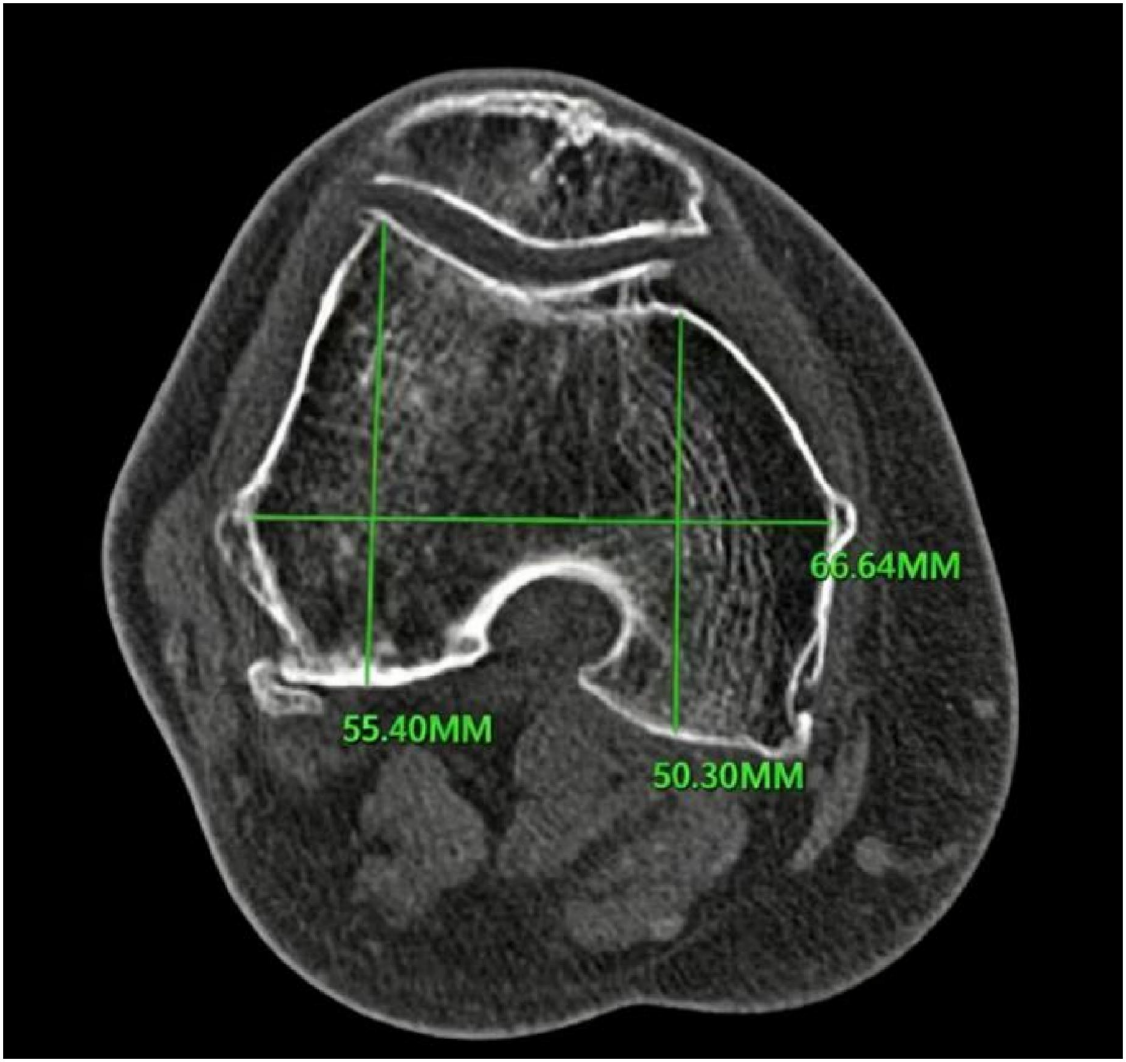
CT-based measurement of distal femoral morphology and aspect ratio. On the axial CT image at the level of the planned distal femoral resection, the mediolateral (ML) diameter was measured parallel to the surgical transepicondylar axis, and the anteroposterior (AP) diameter was measured perpendicular to the ML axis. An additional anteroposterior reference measurement was also recorded for morphological characterization. The aspect ratio was calculated as ML/AP.

On admission, all patients underwent a standardized preoperative evaluation that included a detailed history, physical examination, and routine laboratory workup. Imaging assessment consisted of three components: (1) weight-bearing anteroposterior and lateral radiographs of the affected knee; (2) full-length standing radiographs of both lower extremities to measure the mechanical axis, hip–knee–ankle (HKA) angle, and coronal varus or valgus deformity (Figure 2); and (3) thin-slice computed tomography (CT) with three-dimensional reconstruction to evaluate distal femoral morphology and calculate the ML/AP aspect ratio (Figure 3).

On the full-length standing radiograph, the HKA angle was defined as the angle between the mechanical axis of the femur (line connecting the centre of the femoral head and the centre of the intercondylar notch) and the mechanical axis of the tibia (line connecting the centre of the tibial spines and the centre of the talus).

For CT-based morphometric assessment, measurements were performed on the axial image corresponding to the planned distal femoral resection level. The ML diameter was defined as the maximum width of the distal femur measured parallel to the surgical transepicondylar axis, whereas the AP diameter was defined as the maximum perpendicular distance from the anterior cortex to the posterior condylar line at the level of the lateral femoral condyle. The aspect ratio was calculated as ML/AP.

All preoperative CT and radiographic measurements were performed independently by two senior arthroplasty surgeons with more than 10 years of experience in arthroplasty imaging assessment. To enhance methodological reliability, the measurement protocol was predefined before data collection, and equivocal cases were reviewed in consultation with a musculoskeletal radiologist from our institution. Discrepancies were resolved by consensus. A threshold of 1.13—consistent with the upper end of the aspect ratios offered by commonly used contemporary femoral components (10)—was used to identify patients in whom strict AP referencing would likely result in notable mediolateral under-coverage.

### 2.4 Grouping and femoral component selection strategy

Patients were assigned to one of two groups based on the intraoperative femoral sizing strategy employed by the operating surgeon, which was pre-planned from the CT-based aspect-ratio measurement and verified intraoperatively. Group A (*n* = 52) received a femoral component selected strictly according to the measured AP diameter, following the conventional posterior-referencing technique that is the current mainstream approach in mainland China (11, 18). Group B (*n* = 34) received a femoral component on the basis of the same AP evaluation, with explicit intraoperative criteria: when the preoperative ML/AP ratio exceeded 1.13 and the distal osteotomy surface showed ≥3 mm of uncovered medial or lateral femoral cortex, the surgeon would select a component 1 or 2 sizes larger than the AP-matched size to improve mediolateral coverage. Specifically, in our cohort, 16 patients (47%) were upsized by 1 size and 9 patients (26%) were upsized by 2 sizes, while the remaining 9 patients (27%) ultimately received the AP-matched size after intraoperative adjustment. All femoral components were posterior-stabilized Unique Knee implants provided by Tianjin Zhengtian (Tianjin, China). Demographic variables (age, sex, side) and radiographic parameters (varus angle, femoral ML diameter, AP diameter, initial AP-matched component size, final implanted size, and ML/AP ratio) were recorded for each patient, as summarized in Table 1. In Group B, 47% of patients were upsized by 1 size, 26% by 2 sizes, and 27% received the AP-matched size, providing transparency on the intraoperative sizing decisions.

**Table 1:** Baseline demographic, anatomical, and femoral component sizing characteristics of the two groups.

Variable	Total (*n* = 86)	Group A (*n* = 52)	Group B (*n* = 34)	Statistic	*P* value
Age (years)	65.98 ± 5.76	64.96 ± 5.68	65.00 ± 5.96	*t* = −0.30	0.976
Varus angle (°)	12.33 ± 4.13	11.71 ± 4.17	13.27 ± 3.95	*t*′ = −1.76	0.083
Femoral AP diameter (mm)	56.20 ± 3.45	56.10 ± 3.50	56.35 ± 3.38	*t* = −0.35	0.73
Femoral ML diameter (mm)	65.05 ± 3.92	65.71 ± 3.65	64.03 ± 4.15	*t*′ = 1.92	0.059
ML/AP ratio	1.16 ± 0.05	1.17 ± 0.04	1.15 ± 0.06	*t*′ = 1.88	0.064
Initially AP-matched component size	2.85 ± 0.95	2.73 ± 1.01	2.91 ± 0.88	*t*′ = −1.45	0.152
Final implanted component size	2.92 ± 0.98	2.73 ± 1.01	3.21 ± 0.88	*t*′ = −2.31	0.024
Difference between final and AP-matched size	0.07 ± 0.12	0.00 ± 0.00	0.30 ± 0.25	*t*′ = −4.12	<0.001
AP-matched size retained, *n* (%)	25 (29.1%)	52 (100%)	7 (20.6%)	—	—
Upsized by one size, *n* (%)	34 (39.5%)	0 (0%)	16 (47.1%)	—	—
Upsized by two sizes, *n* (%)	27 (31.4%)	0 (0%)	11 (32.4%)	—	—

AP, anteroposterior; ML, mediolateral.

Data are mean ± standard deviation or *n* (%).

Initially AP-matched component size is the size indicated by conventional posterior-referencing measurement before consideration of ML coverage.

Difference between final and AP-matched size = final implanted size − initially AP-matched size.

Group B represents a selective ML/AP-based sizing strategy rather than a uniformly upsized-component group.

### 2.5 Surgical technique

**Figure 4:**
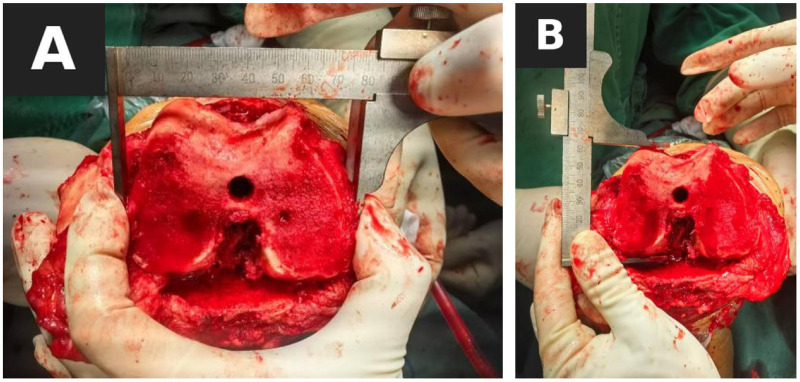
Intraoperative measurement of the distal femoral resected surface. **(A)** The mediolateral (ML) diameter is measured with a calliper placed parallel to the surgical transepicondylar axis. **(B)** The anteroposterior (AP) diameter is measured perpendicular to the ML axis along the lateral femoral condyle.

All operations were performed under general anaesthesia with the patient in the supine position and a pneumatic tourniquet applied to the proximal thigh of the operative limb. A standard midline skin incision with a medial parapatellar arthrotomy was used. After lateral eversion of the patella and debridement of peripatellar soft tissues, osteophytes, and hypertrophic synovium, an intramedullary alignment guide was used to perform distal femoral resection at 5°–7° of valgus. Intraoperative measurement of the femoral ML and AP diameters on the resected distal surface was then performed using a calliper-based sizing jig (Figure 4). Subsequent steps differed between the two groups as follows. (1) In Group A, the femoral component size was determined solely by the AP reading; the four-in-one cutting block was positioned accordingly and the anterior, posterior, and chamfer cuts were performed with the standard posterior-referencing technique. (2) In Group B, the surgeon first evaluated the exposed distal osteotomy surface and, when bare medial or lateral cortex was observed, selected a component one or two sizes larger; the four-in-one cutting block was then translated anteriorly or posteriorly as needed to avoid anterior cortical notching and excessive anterior overhang, and the cutting was performed with the adjusted referencing. In either approach, residual anterior cortical step-off and potential patellofemoral over-stuffing were carefully inspected, and additional anterior soft-tissue release was performed when indicated.

After completion of the femoral preparation, tibial resection was performed with an extramedullary alignment guide, aiming for a neutral mechanical axis in the coronal plane and a posterior tibial slope of approximately 3°–5°. Extension and flexion gaps were assessed with trial components, and soft-tissue releases were performed as needed to achieve symmetrical and balanced gaps. The tourniquet was then released briefly for haemostasis, followed by thorough pulsatile lavage with saline. A standardized peri-articular “cocktail” injection consisting of ropivacaine, adrenaline, and a corticosteroid was administered into the surrounding soft tissues for multimodal analgesia. The tibial and femoral components were then cemented sequentially. The patella was not resurfaced in any patient; instead, peripheral patellar denervation with electrocautery was routinely performed. Final assessment of limb alignment, gap symmetry, patellar tracking, and knee range of motion was carried out, followed by placement of a closed-suction drain, layered wound closure, and sterile compressive dressing.

### 2.6 Postoperative management and rehabilitation

A uniform multimodal perioperative protocol was applied to both groups. Key elements of the perioperative care bundle included the following. (1) Intermittent cryotherapy was applied around the surgical wound for 30 min every 2 h during the first 24 h, and prophylactic intravenous cefuroxime was administered for 24 h postoperatively. (2) Intravenous tranexamic acid 1 g was given at 3 h and again at 6 h after surgery to reduce perioperative and hidden blood loss, in accordance with contemporary evidence supporting multi-dose intravenous regimens in primary TKA (21, 22). (3) The suction drain was clamped for 6 h and usually removed within 24 h; when the 24-h drainage volume exceeded 300 mL, the drain was retained for up to 48 h. (4) Pharmacological venous thromboembolism (VTE) prophylaxis was started 12 h after surgery using subcutaneous enoxaparin 2,000–4,000 U once daily, consistent with current guideline-based practice for hip and knee arthroplasty (23, 24).

Rehabilitation began on the day of surgery. On postoperative day 1, patients were instructed to perform ankle pump exercises, isometric quadriceps contraction, and active straight-leg raising. After removal of the drain, active knee flexion and extension exercises were encouraged, with an early target of 100°–110° of knee flexion; when active flexion was insufficient, continuous passive motion (CPM) and therapist-guided passive mobilization were added. From postoperative day 2 onwards, patients were allowed to ambulate with a walker as tolerated and progressed to partial and then full weight-bearing according to muscle strength and pain tolerance. By postoperative day 7, the target range of motion was 0° to 120°. Most patients returned to unaided walking 6–12 weeks postoperatively.

### 2.7 Follow-up and outcome measures

All patients were followed in the outpatient clinic at 1, 3, 6, and 12 months postoperatively, and annually thereafter; patients unable to attend in-person review were followed by structured telephone interview and review of imaging uploaded remotely. At each follow-up visit, the following data were collected: (1) the Hospital for Special Surgery (HSS) knee score as the primary functional outcome; (2) the Knee Society Score (KSS) as an additional functional measure; (3) the VAS for rest and activity pain; and (4) maximum active knee range of motion measured with a long-arm goniometer. In addition, complications including anterior knee pain, extensor weakness, symptomatic DVT, superficial or deep wound infection, periprosthetic fracture, and component loosening were actively recorded.

Radiographic assessment included standardized standing anteroposterior, lateral, and skyline views at each visit. Particular attention was paid to the distal femoral area not covered by the femoral component. “Radiographic shadowing” was predefined as a focal reduction in trabecular bone density and/or a subtle radiolucent band of less than 2 mm in the uncovered distal femoral condylar region, without radiographic evidence of component loosening or migration.

Postoperative radiographic measurements and interpretation of radiographic shadowing were independently performed by two senior arthroplasty surgeons blinded to group allocation and reviewed according to a predefined imaging assessment protocol. In equivocal cases, the radiographs were further discussed in consultation with a musculoskeletal radiologist from our institution, and the final decision was reached by consensus.

### 2.8 Statistical analysis

Statistical analyses were performed with SPSS version 27.0 (IBM Corp., Armonk, NY, USA). Continuous variables were tested for normality with the Shapiro–Wilk test and are presented as mean ± standard deviation ($\bar{x}\pm s$). Within-group pre- vs. post-operative comparisons were performed with the paired-samples *t* test. Between-group comparisons at a single time point were performed using the independent-samples *t* test or the Welch *t*′ test when variances were unequal. Changes over time (preoperative, 1 week, 3 months, and 1 year) within and between groups were additionally evaluated with repeated-measures analysis of variance. Categorical variables are presented as counts and percentages, and were compared using the *χ*^2^ test or Fisher's exact test where appropriate. A two-tailed *P* value less than 0.05 was considered statistically significant.

## 3 Results

### 3.1 Baseline characteristics

A total of 86 patients (52 in Group A and 34 in Group B) completed at least 12 months of follow-up and were included in the final analysis. Baseline demographic and radiographic characteristics are summarized in Table 1. There were no statistically significant differences between the two groups in mean age, distribution of sex or side, preoperative varus angle, or femoral ML diameter (all *P* > 0.05). As expected by the study design, the mean final implanted femoral component size was larger in Group B than in Group A (3.21 ± 0.88 vs. 2.73 ± 1.01, *t*′ = −2.31, *P* = 0.024). In Group B, 7 patients (20.6%) retained the initially AP-matched size, 16 patients (47.1%) were upsized by one size, and 11 patients (32.4%) were upsized by two sizes, indicating that Group B represents a selective ML/AP-based sizing strategy rather than a uniformly upsized-component group. Preoperative knee ROM, VAS pain score, HSS score, and KSS were comparable between the two groups (Table 2; all *P* > 0.05), indicating that the two cohorts were clinically and functionally similar at baseline.

**Table 2:** Preoperative clinical and functional scores of the two groups.

Variable	Group A (*n* = 52)	Group B (*n* = 34)	*t*	*P* value
Knee ROM (°)	87.69 ± 6.77	87.68 ± 7.91	0.01	0.992
VAS score	6.63 ± 0.86	6.68 ± 0.77	−0.23	0.819
HSS score	57.50 ± 3.74	57.41 ± 4.24	0.1	0.919
KSS score	56.90 ± 3.50	57.10 ± 3.60	−0.34	0.734

ROM, range of motion; VAS, visual analogue scale; HSS, Hospital for Special Surgery score.

### 3.2 Knee range of motion

Both groups showed a marked improvement in knee ROM from baseline to each postoperative time point (all *P* < 0.001). At 1 week postoperatively, Group A achieved a mean ROM of 100.83° ± 5.50°, compared with 96.74° ± 4.19° in Group B, and the between-group difference was statistically significant (*t* = 3.90, *P* < 0.001; Table 3). A similar pattern persisted at 3 months, with Group A showing a larger mean flexion angle than Group B (107.98° ± 5.09° vs. 104.15° ± 4.74°; *t* = 3.56, *P* < 0.001; Table 4). However, by the 1-year follow-up, the ROM difference between the two groups had resolved: Group A reached a mean ROM of 113.15° ± 3.54° and Group B reached 112.44° ± 4.02°, with no statistically significant between-group difference (*t* = 0.86, *P* = 0.390; Table 5).

**Table 3:** Knee range of motion (°): preoperative vs. 1-week comparison.

Preoperative vs. 1-week knee ROM (°)				
Group	Preoperative	1 week	*t*	*P* value
Group A	87.69 ± 6.77	100.83 ± 5.50	−20.10	<0.001
Group B	87.68 ± 7.91	96.74 ± 4.19	−8.46	<0.001
*t*	0.01	3.90*		
*P* value	0.992	<0.001		

* Welch's *t*′ test used due to unequal variances.

**Table 4:** Knee range of motion (°): preoperative vs. 3-month comparison.

Preoperative vs. 3-month knee ROM (°)				
Group	Preoperative	3 months	*t*	*P* value
Group A	87.69 ± 6.77	107.98 ± 5.09	−22.94	<0.001
Group B	87.68 ± 7.91	104.15 ± 4.74	−18.51	<0.001
*t*	0.01	3.56*		
*P* value	0.992	<0.001		

* Welch's *t*′ test used due to unequal variances.

**Table 5:** Knee range of motion (°): preoperative vs. 1-year comparison.

Preoperative vs. 1-year knee ROM (°)				
Group	Preoperative	1 year	*t*	*P* value
Group A	87.69 ± 6.77	113.15 ± 3.54	−30.27	<0.001
Group B	87.68 ± 7.91	112.44 ± 4.02	−21.74	<0.001
*t*	0.01	0.86		
*P* value	0.992	0.390		

No significant between-group difference was observed at the 1-year follow-up.

### 3.3 VAS pain score

VAS pain score improved significantly in both groups at all postoperative time points compared with preoperative values (all *P* < 0.001). In the early postoperative period, Group B reported a slightly higher mean VAS score than Group A. At 1 week, the mean VAS in Group A was 2.90 ± 0.66, vs. 3.35 ± 0.60 in Group B (*t* = −3.26, *P* = 0.002; Table 6). At 3 months, the mean VAS in Group A was 1.38 ± 0.57, vs. 1.82 ± 0.52 in Group B (*t* = −3.69, *P* < 0.001; Table 7). By the 1-year follow-up, both groups had achieved excellent pain relief with very low absolute VAS values (0.08 ± 0.27 in Group A vs. 0.15 ± 0.36 in Group B), and the between-group difference was no longer statistically significant (*t* = −1.03, *P* = 0.304; Table 8). Taken together, these data indicate that although the ML-biased up-sizing strategy was associated with slightly more early-phase pain, the clinical difference was small and transient, and did not persist at mid-term follow-up.

**Table 6:** Pain VAS score: preoperative vs. 1-week comparison.

Preoperative vs. 1-week VAS				
Group	Preoperative	1 week	*t*	*P* value
Group A	6.63 ± 0.86	2.90 ± 0.66	28.89	<0.001
Group B	6.68 ± 0.77	3.35 ± 0.60	20.52	<0.001
*t*	−0.23	−3.26*		
*P* value	0.819	0.002		

* Welch's *t*′ test used due to unequal variances.

**Table 7:** Pain VAS score: preoperative vs. 3-month comparison.

Preoperative vs. 3-month VAS				
Group	Preoperative	3 months	*t*	*P* value
Group A	6.63 ± 0.86	1.38 ± 0.57	39.97	<0.001
Group B	6.68 ± 0.77	1.82 ± 0.52	33.00	<0.001
*t*	−0.23	−3.69*		
*P* value	0.819	<0.001		

* Welch's *t*′ test used due to unequal variances.

**Table 8:** Pain VAS score: preoperative vs. 1-year comparison.

Preoperative vs. 1-year VAS				
Group	Preoperative	1 year	*t*	*P* value
Group A	6.63 ± 0.86	0.08 ± 0.27	55.65	<0.001
Group B	6.68 ± 0.77	0.15 ± 0.36	44.21	<0.001
*t*	−0.23	−1.03		
*P* value	0.819	0.304		

No significant between-group difference in pain VAS at 1 year.

### 3.4 HSS knee score

The HSS knee score showed significant improvement in both groups at every postoperative assessment (all *P* < 0.001). At 1 week postoperatively, Group A had a slightly higher HSS score than Group B (74.77 ± 3.10 vs. 72.00 ± 2.40, *t* = 4.65, *P* < 0.001; Table 9), largely reflecting the earlier regain of ROM observed in Group A. At 3 months (Table 10) and at 1 year (Table 11), HSS scores in the two groups had converged, and the between-group differences were no longer statistically significant (3-month: 81.37 ± 2.16 vs. 80.68 ± 1.72, *t* = 1.56, *P* = 0.122; 1-year: 87.06 ± 2.05 vs. 87.56 ± 2.52, *t* = −1.01, *P* = 0.315). Overall, both groups achieved good-to-excellent mid-term functional scores, consistent with published series of contemporary PS TKA (4, 5).

**Table 9:** HSS knee score: preoperative vs. 1-week comparison.

Preoperative vs. 1-week HSS				
Group	Preoperative	1 week	*t*	*P* value
Group A	57.50 ± 3.74	74.77 ± 3.10	−32.60	<0.001
Group B	57.41 ± 4.24	72.00 ± 2.40	−23.61	<0.001
*t*	0.10	4.65*		
*P* value	0.919	<0.001		

* Welch's *t*′ test used due to unequal variances.

**Table 10:** HSS knee score: preoperative vs. 3-month comparison.

Preoperative vs. 3-month HSS				
Group	Preoperative	3 months	*t*	*P* value
Group A	57.50 ± 3.74	81.37 ± 2.16	−43.40	<0.001
Group B	57.41 ± 4.24	80.68 ± 1.72	−33.52	<0.001
*t*	0.10	1.56		
*P* value	0.919	0.122		

**Table 11:** HSS knee score: preoperative vs. 1-year comparison.

Preoperative vs. 1-year HSS				
Group	Preoperative	1 year	*t*	*P* value
Group A	57.50 ± 3.74	87.06 ± 2.05	−50.55	<0.001
Group B	57.41 ± 4.24	87.56 ± 2.52	−39.00	<0.001
*t*	0.10	−1.01		
*P* value	0.919	0.315		

### 3.5 Operative parameters and complications

There were no statistically significant differences between the two groups in operative time, intraoperative blood loss, or postoperative drainage (all *P* > 0.05). During the follow-up period, two patients in Group A and one patient in Group B developed an intramuscular calf venous thrombosis, all of which were managed conservatively with low-molecular-weight heparin and resolved without sequelae (*P* > 0.05). No case of symptomatic pulmonary embolism, deep surgical site infection, periprosthetic fracture, or aseptic loosening was observed in either group. Neither group showed clinically relevant extensor weakness at final follow-up, and anterior knee pain of any grade was infrequent in both groups and did not differ significantly.

### 3.6 Radiographic shadowing in the uncovered distal femoral region

**Figure 5:**
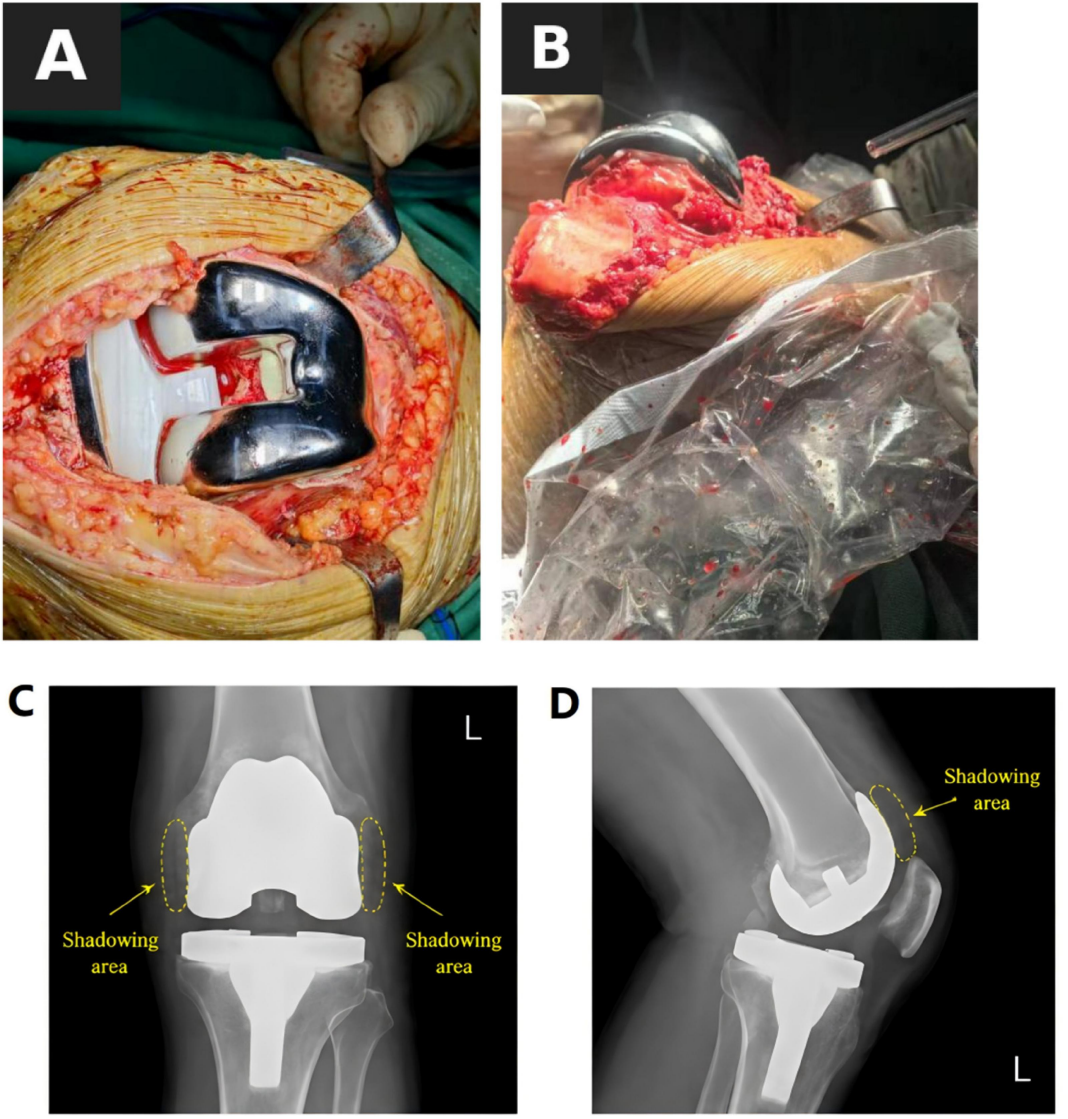
Representative intraoperative and radiographic findings of the uncovered distal femoral region. **(A,B)** Intraoperative views showing the uncovered distal femoral condylar region after trial component implantation. **(C,D)** Annotated postoperative anteroposterior and lateral radiographs showing focal reduction in bone density or subtle radiolucency in the uncovered distal femoral region, indicated by arrows.

Radiographic review at 3 months revealed a markedly different pattern of bone adaptation in the uncovered distal femoral region between the two groups. In Group A, 34 of 52 knees (65.38%) exhibited radiographic shadowing—defined as a focal reduction in trabecular bone density with subtle radiolucency in the medial or lateral distal femoral area not covered by the femoral component—whereas only 7 of 34 knees (20.59%) in Group B showed similar findings. These radiographic changes corresponded to the intraoperative pattern of distal femoral under-coverage illustrated in Figures 5A,B. Annotated postoperative anteroposterior and lateral radiographs highlighting the areas of reduced bone density or subtle radiolucency are presented in Figures 5C,D. The between-group difference was highly significant (*χ*^2^ = 16.54, *P* < 0.001; Table 12).

**Table 12:** Incidence of radiographic shadowing in the uncovered distal femoral region.

Radiographic shadowing in the uncovered area	Group A, *n* (%)	Group B, *n* (%)	*χ* ^2^	*P* value
No	18 (34.62)	27 (79.41)	16.54	<0.001
Yes	34 (65.38)	7 (20.59)		

Data are *n* (%). Assessed at 3-month postoperative radiographic follow-up.

## 4 Discussion

The central finding of this retrospective cohort study is that, in Chinese patients with primary knee osteoarthritis and an ML/AP ratio greater than 1.13, deliberate upsizing of the femoral component by one or two sizes on the basis of the mediolateral dimension substantially reduced radiographic shadowing in the uncovered distal femoral region (from 65.38% to 20.59%; *P* < 0.001) without compromising one-year functional scores or pain scores. The early transient disadvantage of the ML-biased strategy—slightly less ROM and slightly higher VAS during the first three postoperative months—is a clinically relevant observation that surgeons should acknowledge, but it did not translate into inferior mid-term outcomes. These findings complement the contemporary literature on femoral component fit in Asian TKA and provide pragmatic guidance for intraoperative decision-making.

TKA for end-stage KOA continues to deliver reliable and durable pain relief and functional improvement. Contemporary single-centre series report ten-year survivorship of 94.1% with modern morphometric PS designs and high patient satisfaction (4, 5). Nevertheless, a persistent 10%–20% of patients report dissatisfaction, and anterior knee pain together with residual patellofemoral discomfort remain leading non-infectious reasons for revision (6, 7). Optimizing implant fit is therefore a principal target for improvement, and the mismatch between off-the-shelf femoral components and the morphology of individual patients has been repeatedly implicated in suboptimal outcomes (8–10, 19). A systematic review by Bonanzinga et al. found that, across four commonly used contemporary off-the-shelf femoral designs, 13%–41% of patients would experience clinically relevant underhang and 9%–27% overhang when implants were applied to anthropometric datasets (8). The morphological mismatch is particularly pronounced in female and Asian knees, whose distal femora are generally smaller and have a different aspect ratio than those of the Caucasian populations from which most implants were designed (10, 16).

Because TKA was developed in Europe and North America, the implant inventory available in China historically reflected Western morphology, with a standard ML/AP aspect ratio of approximately 1.02–1.13 (10). Morphometric studies in Chinese osteoarthritic knees, including a recent CT-based analysis of 406 knees from southwestern China (10), have confirmed that a substantial proportion of patients have aspect ratios above this range, especially among women. Although several Chinese manufacturers have begun to refine their designs with wider ML offerings, a considerable number of patients still present intraoperatively with an ML diameter that exceeds the width of the AP-matched component. The present study, which used a single PS design in a uniform surgical team, illustrates how this anatomical reality can be addressed with a reproducible intraoperative decision rule.

In Group A, where the femoral component was selected strictly according to the AP diameter, the early clinical results were favourable, with excellent ROM and low pain scores at 1 week. This is consistent with meta-analytical data showing that anterior or posterior referencing yield comparable early clinical outcomes despite different effects on PCO (11). However, radiographic follow-up revealed that 65.38% of Group A knees had a zone of reduced bone density in the uncovered distal medial or lateral femoral condyle, with larger uncovered areas showing more severe changes. Although such radiographic findings are not equivalent to clinical failure, they suggest altered local stress distribution and possible stress shielding, which has been associated with long-term concerns including late loosening and periprosthetic fracture (9, 15). Whether these early radiographic changes will translate into clinically relevant complications remains to be defined by longer follow-up.

In Group B, selecting a component one or two sizes larger to improve ML coverage introduced a trade-off. A larger component modestly increases the risk of ML overhang, increases patellofemoral stuffing, and may slightly tighten the anterior flexion envelope; in our cohort this manifested as moderately reduced ROM and higher VAS during the first three postoperative months. In practice, the surgeon could compensate for part of this effect by translating the four-in-one cutting block to adjust anterior–posterior positioning and, in selected cases, accepting slight sacrifice of the flexion gap in order to limit patellofemoral compression. Importantly, by 1-year follow-up, both ROM and VAS scores had equalized between the two groups, and HSS scores were comparable. This pattern echoes the systematic review by Bonnin et al., which reported that moderate ML oversizing can adversely affect early flexion and pain, but does not preclude reaching satisfactory outcomes when rehabilitation is optimized (19). Our data suggest that, in Asian knees with a high ML/AP ratio, the early disadvantage of up-sizing is outweighed by the reduction in distal femoral under-coverage.

The mechanism underlying the radiographic shadowing we observed in Group A is likely multifactorial and relates to altered local bone stress. When the medial or lateral distal femoral cortex remains uncovered, the bone surface is no longer supported by the metallic component and experiences a different loading pattern, which may lead to disuse atrophy and resorption in the short term (8, 9). Some of these changes appeared to reverse partially at 1-year follow-up, presumably as bone remodels to the new load environment, but a sizeable subset of patients retained the radiographic pattern. Long-term prospective imaging is required to determine whether these changes correlate with late aseptic loosening, periprosthetic fracture, or component sinkage—outcomes that were not observed within our one-year follow-up window but that are well known complications of TKA with long follow-up (7, 9). The balance between component coverage and ML overhang is therefore not merely a short-term technical choice but a determinant of long-term implant survivorship.

The role of PCO also deserves mention. Multiple studies have confirmed that PCO restoration is associated with better postoperative flexion, and that anterior referencing tends to reduce PCO while posterior referencing tends to preserve or increase it (11, 18). When up-sizing the femoral component in Group B, careful re-positioning of the cutting block is essential to avoid inadvertent reduction of PCO in the sagittal plane. In our series, both groups achieved satisfactory flexion at 1 year, suggesting that meticulous gap balancing during the four-in-one cut can reconcile ML up-sizing with adequate PCO preservation. Nevertheless, surgeons should remain vigilant, as inappropriate PCO reduction may produce flexion instability or mid-flexion pain that is difficult to salvage (11).

Anterior knee pain remains a common concern after TKA and is the leading non-infectious cause of revision in contemporary series (7). Because ML up-sizing increases patellofemoral stuffing, one might expect a higher incidence of anterior knee pain in Group B. In our cohort, however, anterior knee pain was infrequent in both groups and did not differ significantly. Several factors may explain this result, including the consistent use of peripatellar denervation, careful avoidance of excessive anterior overhang by selective anterior translation of the cutting block, and preservation of patellar thickness through non-resurfacing of the patella. Our findings are consistent with a recent retrospective study showing that preoperative patellofemoral alignment parameters—rather than implant sizing *per se*—are the strongest predictors of postoperative anterior knee pain in TKA without patellar resurfacing (25).

Several additional aspects of the current technique trend deserve discussion. The rapid adoption of robot-assisted TKA promises more precise and reproducible bone cuts and component positioning, and multiple studies have shown improved alignment accuracy compared with conventional techniques (26, 27). However, most commercial robotic systems inherit the same AP-referencing philosophy and implant inventory as their conventional counterparts, and therefore may replicate the under-coverage problem we observed in Group A when applied to Asian knees. A recent analysis in fact showed that robotic-assisted TKA tends to select smaller femoral components than the manual technique (27). As the Chinese population continues to develop regional variability in body size and knee morphology, the design of implants and surgical planning software should explicitly account for the locally observed distribution of aspect ratios. Whether the standard ML/AP range of 1.02–1.13 remains appropriate for China is an open question that warrants further multicentre morphometric work (10, 28).

Perioperative management has converged around multimodal protocols in recent years. The use of multi-dose intravenous tranexamic acid (21, 22), early mobilization with ankle pump and straight-leg raise exercises, and chemoprophylaxis with low-molecular-weight heparin following international arthroplasty guidelines (23, 24) all played roles in achieving the low complication rate observed in this cohort. The incidence of calf intramuscular vein thrombosis in our series (2/52 in Group A and 1/34 in Group B) was comparable to that of recent large series and was not significantly affected by the sizing strategy (23, 24). These results support the conclusion that the observed between-group differences reflect the sizing decision itself rather than confounding by perioperative care.

This study has several limitations. First, the retrospective single-centre design and relatively modest sample size (86 patients, 34 in the ML-biased upsizing group) limit the precision of our estimates and the external generalizability of our findings. However, the detailed reporting of individual intraoperative sizing decisions, including the proportion of patients upsized by 1 or 2 sizes vs. those who remained AP-matched, enhances reproducibility and provides a practical guide for similar Asian patient populations with high ML/AP ratios. Second, the follow-up of one year is short for a discussion of implant survivorship and long-term periprosthetic bone remodelling; continued observation is required to determine whether the radiographic shadowing seen in Group A will translate into late loosening or fracture. Third, although all procedures were performed by the same surgical team using a uniform technique and a single PS implant, allocation to Group A or Group B was determined by surgeon judgement rather than randomization, which introduces selection bias. Fourth, intraoperative measurement of the ML and AP diameters was based on calliper-assisted estimation, which, though clinically pragmatic, is less accurate than computer-assisted three-dimensional measurement. Finally, this study evaluated patellar-specific outcomes only by VAS for anterior knee pain and clinical examination, without dedicated patellofemoral scores. Future research should incorporate these measures, as well as patient-reported outcome measures (PROMs) and subjective satisfaction scores, to better capture the patient's perspective on functional recovery and quality of life. Despite these limitations, this study provides pragmatic and clinically relevant evidence that informs femoral sizing decisions in Asian patients with a high ML/AP ratio.

## 5 Conclusion

TKA remains an effective treatment for end-stage knee osteoarthritis, providing durable pain relief and functional improvement in both sizing groups in this study. In Asian patients with an ML/AP ratio greater than 1.13, choosing a femoral component 1–2 sizes larger than that predicted by strict AP referencing appropriately increases distal mediolateral coverage and reduces the incidence of radiographic shadowing in the uncovered distal femoral area, at the cost of a modest and transient decrease in ROM and slight increase in VAS during the first three postoperative months, without differences at one year. This ML-informed up-sizing strategy merits consideration in routine surgical practice for Asian populations, and the optimal aspect-ratio offering of future implants designed for this population warrants continuing investigation.

## Data Availability

The original contributions presented in the study are included in the article/Supplementary Material, further inquiries can be directed to the corresponding author.

## References

[1] Katz JN Arant KR Loeser RF. Diagnosis and treatment of hip and knee osteoarthritis: a review. JAMA. (2021) 325(6):568–78. 10.1001/jama.2020.22171 33560326 PMC8225295

[2] Langworthy M Dasa V Spitzer AI. Knee osteoarthritis: disease burden, available treatments, and emerging options. Ther Adv Musculoskelet Dis. (2024) 16:1759720X241273009. 10.1177/1759720X241273009 39290780 PMC11406648

[3] Long H Liu Q Yin H Wang K Diao N Zhang Y. Prevalence trends of site-specific osteoarthritis from 1990 to 2019: findings from the global burden of disease study 2019. Arthritis Rheumatol. (2022) 74(7):1172–83. 10.1002/art.42089 35233975 PMC9543105

[4] Siret E Sammartino F Bernard De Villeneuve F Nasser Al Maeen B Argenson JN Jacquet C. Minimum ten years follow-up of total knee arthroplasty using morphometric implants in patients with osteoarthritis. Int Orthop. (2026) 50(2):393–400. 10.1007/s00264-025-06703-0 41288690

[5] Singh M Harary J Schilling PL Moschetti WE. Patient satisfaction is nearly 90% after total knee arthroplasty; we are better than we were. J Arthroplasty. (2025) 40(6):1521–5.e1. 10.1016/j.arth.2024.11.038 39581239

[6] Kahlenberg CA Nwachukwu BU McLawhorn AS Cross MB Cornell CN Padgett DE. Patient satisfaction after total knee replacement: a systematic review. HSS J. (2023) 19(1):1–8. 10.1007/s11420-018-9614-8 PMC6031540 29983663

[7] Upfill-Brown A Hsiue PP Sekimura T Shi B Ahlquist SA Patel JN. Epidemiology of revision total knee arthroplasty in the United States, 2012 to 2019. Arthroplast Today. (2022) 15:188–95. 10.1016/j.artd.2022.03.004 35774881 PMC9237286

[8] Bonanzinga T Gambaro FM Iacono F Marcacci M. Sub-optimal femoral fit in total knee arthroplasty: a systematic review of human femoral data vs off-the-shelf contemporary femoral components. J Exp Orthop. (2023) 10(1):41. 10.1186/s40634-023-00607-x 37036541 PMC10086082

[9] Bernard-de Villeneuve F Bizzozero P Fabre-Aubrespy M Ollivier M Argenson J-N. Does matching femoral size and shape improve bone fit and patient-reported outcomes in TKA? A matched controlled study. Clin Orthop Relat Res. (2023) 481(6):1129–39. 10.1097/CORR.0000000000002530 36716085 PMC10194518

[10] Ke S Ran T Ma T Qin Y Zhang B Wang M. A morphometric study of the distal femoral resected surface in osteoarthritis knees of the patients in southwest China and a comparison with femoral components in six total knee arthroplasty systems. Orthop Surg. (2023) 15(4):953–60. 10.1111/os.13647 36718658 PMC10102296

[11] Familiari F Mercurio M Napoleone F Galasso O Giuzio E Simonetta R. Anterior referencing versus posterior referencing in primary total knee arthroplasty: a meta-analysis of randomized controlled trials. J Clin Med. (2023) 12(23):7453. 10.3390/jcm12237453 38068504 PMC10707523

[12] Dai Y Scuderi GR Penninger C Bischoff JE Rosenberg A. Increased shape and size offerings of femoral components improve fit during total knee arthroplasty. Knee Surg Sports Traumatol Arthrosc. (2014) 22(12):2931–40. 10.1007/s00167-014-3163-6 25026932 PMC4237918

[13] Kwak DS Han S Han CW Han SH. Resected femoral anthropometry for design of the femoral component of the total knee prosthesis in a Korean population. Anat Cell Biol. (2010) 43(3):252–9. 10.5115/acb.2010.43.3.252 21212865 PMC3015043

[14] Mohan H Chhabria P Bagaria V Tadepalli K Naik L Kulkarni R. Anthropometry of nonarthritic Asian knees: is it time for a race-specific knee implant? Clin Orthop Surg. (2020) 12(2):158–65. 10.4055/cios19069 32489536 PMC7237262

[15] Flores CL San Juan JAG. Morphometric analysis of the Filipino knee and its implication in total knee arthroplasty prosthesis design. Arthroplasty. (2022) 4(1):15. 10.1186/s42836-022-00117-8 35379359 PMC8981831

[16] Wang B Zhang G Pu R Li Q Wang Y. Clinical significance of distal femur morphology in a healthy Mongolian youth population. Sci Rep. (2023) 13(1):8187. 10.1038/s41598-023-35463-3 37210457 PMC10199924

[17] Prithishkumar IJ Abdulwahab T Mohammad A Albelooshi A. Estimating aspect ratio of the distal femur and proximal tibia in the Emirati population from MRI scans of the knee: a preliminary experience. Sci Rep. (2023) 13(1):4489. 10.1038/s41598-023-31715-4 36934199 PMC10024718

[18] Panciera A Digennaro V Bogucki BDB Barile F Manzetti M Ferri R. The role of posterior condylar offset ratio on clinical and functional outcome of posterior stabilized total knee arthroplasty: a retrospective cohort study. Eur J Orthop Surg Traumatol. (2023) 33(6):2459–64. 10.1007/s00590-022-03459-w 36536107

[19] Van de Kelft A De Mulder K De Schepper J Victor J Vundelinckx B. Balancing the flexion gap first in total knee arthroplasty leads to better preservation of posterior condylar offset resulting in better knee flexion. Knee Surg Sports Traumatol Arthrosc. (2023) 31(9):3792–8. 10.1007/s00167-023-07346-7 36809510

[20] Nojiri S Hayakawa K Date H Naito Y Sato K Uraya Y. Which is better? Anterior or posterior referencing for femoral component position in total knee arthroplasty. J Orthop Surg (Hong Kong). (2021) 29(1):23094990211002325. 10.1177/23094990211002325 33779392

[21] Kirwan MJ Diltz ZR Dixon DT Rivera-Peraza CA Gammage CJ Mihalko WM. The AAHKS clinical research award: extended postoperative oral tranexamic acid in total knee arthroplasty: a randomized controlled pilot study. J Arthroplasty. (2024) 39(8 Suppl):S13–17. 10.1016/j.arth.2024.02.073 38430972

[22] Maccagnano G Pesce V Noia G Coviello M Vicenti G Vitiello R. The effects of a new protocol on blood loss in total knee arthroplasty. Orthop Rev (Pavia). (2022) 14(5):37625. 10.52965/001c.37625 36035591 PMC9404250

[23] Cheok T Beveridge A Berman M Coia M Campbell A Tse TTS. Efficacy and safety of commonly used thromboprophylaxis agents following hip and knee arthroplasty. Bone Joint J. (2024) 106-B(9):924–34. 10.1302/0301-620X.106B9.BJJ-2023-1252.R2 39216864

[24] Lan R Stiles ER Ward SA Lajam CM Bosco JA. Trends in deep vein thrombosis prophylaxis after total knee arthroplasty: 2016 to 2021. J Arthroplasty. (2024) 39(7):1736–41. 10.1016/j.arth.2024.01.039 38325530

[25] Kim SH Kang K-T Koh J-H Park Y-B Lee H-J. Preoperative patello-femoral alignment affects anterior knee pain after primary total knee arthroplasty without patellar resurfacing. J Arthroplasty. (2025) 40(6):1554–9. 10.1016/j.arth.2024.11.042 39581237

[26] Lee YM Kim GW Lee CY Song E-K Seon J-K. No difference in clinical outcomes and survivorship for robotic, navigational, and conventional primary total knee arthroplasty with a minimum follow-up of 10 years. Clin Orthop Surg. (2023) 15(1):82–91. 10.4055/cios21138 36779002 PMC9880514

[27] Bernstein J Hepinstall M Donnelley C Rajahraman V Waren D Schwarzkopf R. Robotic arm–assisted total knee arthroplasty results in smaller femoral components and larger tibial baseplates than the manual technique. Arthroplast Today. (2024) 29:101414. 10.1016/j.artd.2024.101414 39529977 PMC11551327

[28] Khow YZ Liow MHL Lee M Chen JY Lo NN Yeo SJ. Posterior condylar offset and posterior tibial slope targets to optimize knee flexion after unicompartmental knee arthroplasty. Knee Surg Sports Traumatol Arthrosc. (2022) 30(3):948–58. 10.1007/s00167-021-06453-7 33512542

